# Quantitative Analyses of the Left Ventricle Volume and Cardiac Function in Normal and Infarcted Yucatan Minipigs

**DOI:** 10.3390/jimaging7070107

**Published:** 2021-07-01

**Authors:** Anna V. Naumova, Gregory Kicska, Kiana Pimentel, Lauren E. Neidig, Hiroshi Tsuchida, Kenta Nakamura, Charles E. Murry

**Affiliations:** 1 Department of Radiology, University of Washington, Seattle, WA 98109, USA; kicskag@uw.edu (G.K.); pimentelkiana00@gmail.com (K.P.); 2 Institute for Stem Cells and Regenerative Medicine, University of Washington, Seattle, WA 98109, USA; lneidig@uw.edu (L.E.N.); tsuchida@uw.edu (H.T.); nakamur@cardiology.washington.edu (K.N.); murry@uw.edu (C.E.M.); 3 Department of Pathology, University of Washington, Seattle, WA 98109, USA; 4 Department of Comparative Medicine, University of Washington, Seattle, WA 98109, USA; 5 Division of Cardiology, Department of Medicine, University of Washington, Seattle, WA 98195, USA

**Keywords:** left ventricular chamber volume, heart contractility, papillary muscles, animal models

## Abstract

(1) Background: The accuracy of the left ventricular volume (LVV) and contractility measurements with cardiac magnetic resonance imaging (CMRI) is decreased if the papillary muscles are abnormally enlarged, such as in hypertrophic cardiomyopathy in human patients or in pig models of human diseases. The purpose of this work was to establish the best method of LVV quantification with CMRI in pigs. (2) Methods: The LVV in 29 Yucatan minipig hearts was measured using two different techniques: the “standard method”, which uses smooth contouring along the endocardial surface and adds the papillary volume to the ventricular cavity volume, and the “detailed method”, which traces the papillary muscles and trabeculations and adds them to the ventricular mass. (3) Results: Papillary muscles add 21% to the LV mass in normal and infarcted hearts of Yucatan minipigs. The inclusion or exclusion of these from the CMRI analysis significantly affected the study results. In the normal pig hearts, the biggest differences were found in measurements of the LVV, ejection fraction (EF), LV mass and indices derived from the LV mass (*p* < 0.001). The EF measurement in the normal pig heart was 11% higher with the detailed method, and 19% higher in the infarcted pig hearts (*p* < 0.0001). The detailed method of endocardium tracing with CMRI closely represented the LV mass measured *ex vivo*. (4) Conclusions: The detailed method, which accounts for the large volume of the papillary muscles in the pig heart, provides better accuracy and interobserver consistency in the assessment of LV mass and ejection fraction, and might therefore be preferable for these analyses.

## 1. Introduction

Cardiac magnetic resonance imaging (CMRI), with its high spatial and contrast resolution, especially in soft tissues is considered the gold standard for the non-invasive assessment of the heart structure and contractility. CMRI has a high accuracy in the evaluation of the left ventricle volume (LVV) and mass (LV mass) in humans as well as in various experimental animal models [1,2,3,4,5]. Assessment of the LVV is typically done using multiple 2-dimensional short axis slices spanning the heart acquired at the cine mode. The semi-automated image segmentation tools implemented in commercially available software packages help to streamline workflows for clinical applications.

Due to the superior image resolution and high contrast between the blood pool and myocardial tissue, the papillary muscles and even the trabeculae of the left ventricle are easily identified in cine MRI. The anatomy of papillary muscles and trabeculations are complicated [6,7]. The normal papillary muscle mass in humans has been reported to be 13.5 ± 4.1 g [8]; that value doubles in patients with hypertrophic cardiomyopathy [9]. Based on the literature, the papillary muscles can account for up to 8.9–10.5% of the total LV mass in normal humans [8,10], and up to 5–7% of the total LV mass in dogs and pigs [4,11]. A study reported by Peters et al. [12] showed the trabecular-papillary muscle complex, visible in CMRI as a mixture of blood and endocardial structures, constitutes as much as 50% of the myocardial wall in some sectors; in low resolution imaging, this will lead to a 100% overestimation of the end-diastolic wall thickness. The left ventricular papillary muscles are the last portions of the heart to be perfused by coronary arterial blood; therefore, they are sensitive anatomic markers of myocardial ischemia [7]. Considering all of the above, the inclusion or exclusion of papillary muscles and trabeculae in the LV mass will likely affect the cardiac volume, mass, and function measurements.

The standard method of LV tracing that is widely used clinically is drawing smooth contouring (circles) for partitioning the ventricular myocardium from the ventricular cavity, which includes the myocardial papillary and trabecular muscles in the ventricular cavity volume and exclude those from the ventricular mass. This method is relatively fast and diagnostically accurate for the estimation of heart geometry and LV mass in the majority of clinical cases [13,14]. The accuracy of left ventricular volume and contractility measurements is decreased if trabeculations or papillary muscles are abnormally enlarged, such as in hypertrophic cardiomyopathy (HCM) [10]. Inaccuracies in ventricular volume estimates impact the calculation of mitral regurgitation volume, which is an important clinical variable in these patients [15]. In HCM patients, the LVV and heart contractility measurements are more accurate if the contours of the trabecular and papillary muscles are traced and included into the LV mass volume [15].

**Figure 1:**
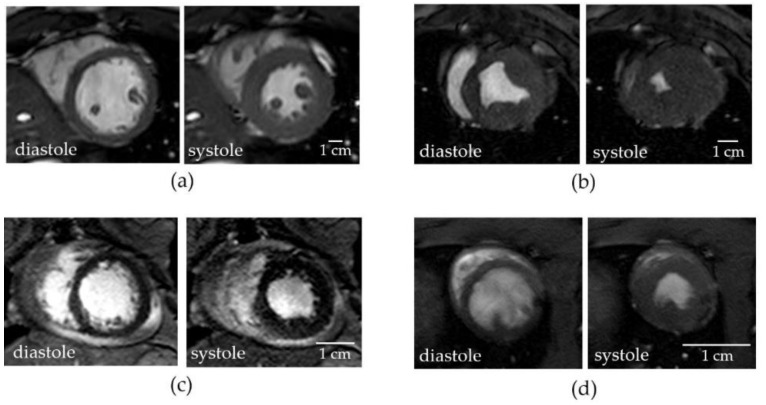
Representative short axis MR images of a human heart, (**a**) and hearts of laboratory animals: pig (**b**), non-human primate (**c**), and rat hearts (**d**), acquired at the end-systolic and end-diastolic phase of the cardiac cycle. CMRI exams were performed using the 3T Philips whole body scanner at the BioMolecular Imaging Center (University of Washington, Seattle, WA, USA). In pigs (**b**), massive papillary muscles often obliterate the entire left ventricle cavity. The heart of non-human primates (**c**) has smaller papillary muscles with an extensive trabecular net.

Large and small laboratory animals are often used as models of human cardiovascular diseases and for the evaluation of therapeutic interventions. The hearts of different animal species have specific anatomical features, and different volumes and geometries of the papillary and trabecular muscles (examples are in Figure 1). Pigs and non-human primates are often used in the pre-clinical testing of therapeutic interventions because of their similarity to human physiology. However, pigs have relatively large papillary muscles and trabeculae (Figure 1b); therefore, standard methods of assessment of the heart LVV and contractile function might carry the same problems as HCM patients, and the accuracy of CMRI measurements in pigs might be affected by the tracing method that was applied. There are only a few publications exploring the role of papillary muscles and trabeculae in the LVV, LV mass, and the heart contractility assessment in large animals. Therefore, there is a need to establish the best method of contouring in commonly used animal models of human diseases. To address this need, we determined the most precise contouring method in infarcted and normal pig hearts by comparing the LVV and contractile measurements using contours that either include or exclude the papillary muscles.

## 2. Materials and Methods

### 2.1. Animals

Twenty-nine castrated male, young adult, Yucatan minipigs weighing between 30–40 kg were included in the study. All procedures and protocols were approved and conduced in accordance with the University of Washington (UW) Institutional Animal Care and Use Committee (IACUC). Pigs were housed in the facilities of the UW Department of Comparative Medicine, which are in compliance with the principles of the “Guide for Laboratory Animal Facilities and Care” of the National Academy of Sciences, National Research Council. The pigs were under the care of UW staff veterinarians with extensive experience in large animal health in consultation with clinical cardiologists. Animals received ad libitum water and were fed twice a day (Lab Diet—Porcine Grower Diet). Additionally, the animals had a minimum 5-day acclimation period before being enrolled in the study.

### 2.2. Myocardial Infarction

For surgical procedures, the animals were sedated with a combination of Butorphanol, Acepromazine and Ketamine, which was administered intramuscularly. The animals were intubated and mechanically ventilated using Isoflurane and oxygen to maintain a surgical plane of anesthesia. Vital signs were measured continuously throughout each procedure. All anesthetic and surgical procedures were performed under the care and supervision of a veterinarian or licensed veterinary technician. All animals received Buprenorphine SR-Lab (ZooPharm) for post-operative analgesia after surgical procedures. Myocardial infarction (MI) was modeled using percutaneous ischemia/reperfusion [16]. Briefly, a guiding catheter was used to engage the left coronary artery and then a percutaneous transluminal coronary angioplasty (PTCA) balloon catheter (2.5 mm/8 mm, ApexTM monorail balloon catheter (Boston Scientific, Marlborough, MA, USA) was used to occlude blood flow distal to the first diagonal branch of the left anterior descending coronary artery (LAD), followed by reperfusion. LAD occlusion was confirmed by angiography and ECG changes (ST segment elevation in ECG).

### 2.3. Cardiac Magnetic Resonance Imaging (CMRI)

In vivo CMRI studies were conducted on a 3T Ingenia CX clinical scanner (Philips, Best, The Netherlands) at different time points: on healthy animals before MI modeling, and then at 2, 4, 8, 12, and 16 weeks after MI. During the scan, the animals were sedated with a combination of Butorphanol, Acepromazine and Ketamine, administered intramuscularly. Animals were then intubated and mechanically ventilated using Isoflurane and oxygen to maintain a surgical plane of anesthesia during the scan. Vital signs were measured continuously throughout each procedure.

For assessment of the LVV and contractility, cine CMRI acquisitions were used with a balanced turbo field echo (bTFE) sequence that generated ~30 cardiac phases for 12 short axis slices spanning the left ventricle. Acquisition parameters included a repetition time (TR) of 3.5 ms, an echo time (TE) of 1.8 ms, a 45° flip angle (FA), a field of view of 250 × 250 mm, a slice thickness of 6 mm with no gaps, an in-plane resolution of 1.3 × 1.3 mm, and one signal average. All acquisitions were ECG-gated with breath-hold.

For quantification of the infarct size, minipigs subsequently received an intravenous injection of the Gd-based contrast agent ProHance (0.2 mmol/kg, Bracco Diagnostics Inc., Princeton, NJ, USA) bolus followed by a saline flush. Late gadolinium enhanced (LGE) images were acquired at the short axis of the heart with an ECG-gated, breath-hold, phase-sensitive inversion recovery (PSIR) sequence 10 min following the contrast agent injection [17]. The inversion time (TI) was adjusted by the scanner operator after the look-locker acquisition to null signal from the non-infarcted remote myocardium. PSIR acquisition parameters included a TR of 7.1 ms, a TE of 3.5 ms, a FA of 25°, a TI range 280–350 ms, a field of view of 250 × 250 mm, a slice thickness of 6 mm without a gap between slices, and one signal average. PSIR images were acquired at the mid-diastolic phase of the cardiac cycle.

After the last CMRI, animals were maintained under anesthesia and then euthanized by intravenous administration of a commercially available veterinary approved euthanasia solution (Euthasol) containing pentobarbital sodium and phenytoin sodium. Euthanasia procedures were performed in compliance with the AVMA Guidelines for the Euthanasia of Animals: 2020 Guidelines. Hearts were extracted, blood was washed out of the heart chambers, and the right atrium and right ventricle were removed. The left ventricle was weighted *ex vivo*. Since the imaged animals were part of the other larger study, in some cases hearts were explanted not on the same day after the CMRI exam, but in later time points.

### 2.4. Image Analysis

**Figure 2:**
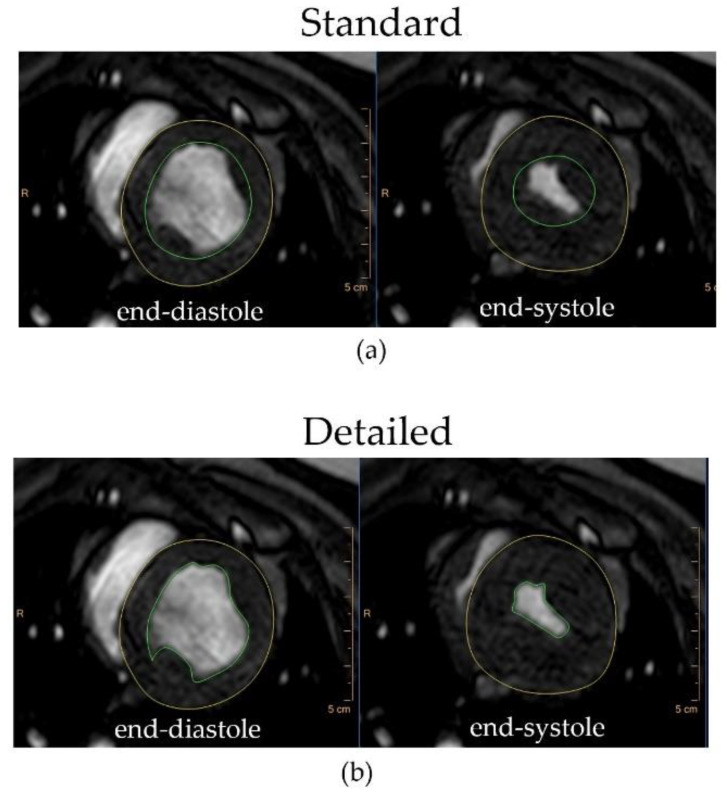
Examples of the standard (**a**) and detailed (**b**) tracing methods of the normal Yucatan minipig heart at the end-systolic and end-diastolic phases of the cardiac cycle.

A total of 69 CMRI cases were analyzed: 29 normal pig hearts and 40 infarcted hearts. Hearts of infarcted pigs were imaged at these different time points after myocardial infarction (MI): 16 animals at 2 weeks after MI; 7 animals at 4 weeks; 7 pigs at 8 weeks; 6 animals at 12 weeks; 4 pigs at 16 weeks. Two reviewers conducted the measurements using Philips IntelliSpace Portal (ISP) software. LV epicardial and endocardial boundaries were interactively traced at end-diastole and end-systole to obtain the end-systolic volume (ESV), end-diastolic volume (EDV), LV end-diastolic mass (LVmass), cardiac output (CO) and the LV ejection fraction (EF). The volumetric analysis of the heart was performed by two techniques: the standard method of smooth contouring (circles) for partitioning the ventricular myocardium from the ventricular cavity that includes the myocardial papillary and trabecular muscles in the ventricular cavity volume, and the detailed method, which included endocardial trabeculations and papillary muscles in the ventricular mass (Figure 2). The infarct size was measured from the PSIR multislice images using both the standard and the detailed tracing method and presented as a percentage of the scar tissue to LV mass.

### 2.5. Statistical Analysis

Microsoft Excel (Microsoft Inc., Redmond, WA, USA) and SPSS 12.0 (IBM Inc., Armonk, NY, USA) software were used for data analysis. Results are presented as mean ± standard deviation (SD). The paired samples *t*-test was used to verify the differences in heart contractile parameters between the standard and detailed measurement methods and for comparisons of LVV and contractility changes in the same animals before and after infarction. Longitudinal changes were compared using the *t*-test for independent samples because a different number of animals were used in each time point and some animals were sacrificed at each time point. Linear regression analysis was used to find relations between the CMRI results derived with the use of the standard and detailed tracing methods and to find relations between *in vivo* and *ex vivo* assessments of LV mass. The Pearson correlation and Bland–Altman analysis methods were used to compare correspondence in measurements between the two methods and between reviewers [18,19]. The mean difference and standard deviation (SD) were reported for the Bland–Altman analysis. Interclass correlation was used to assess the reliability of measurements [20]. The results were considered statistically significant if *p* values were smaller than 0.05. 

## 3. Results

### 3.1. Volumetric LV Analysis in the Normal Pigs

**Figure 3:**
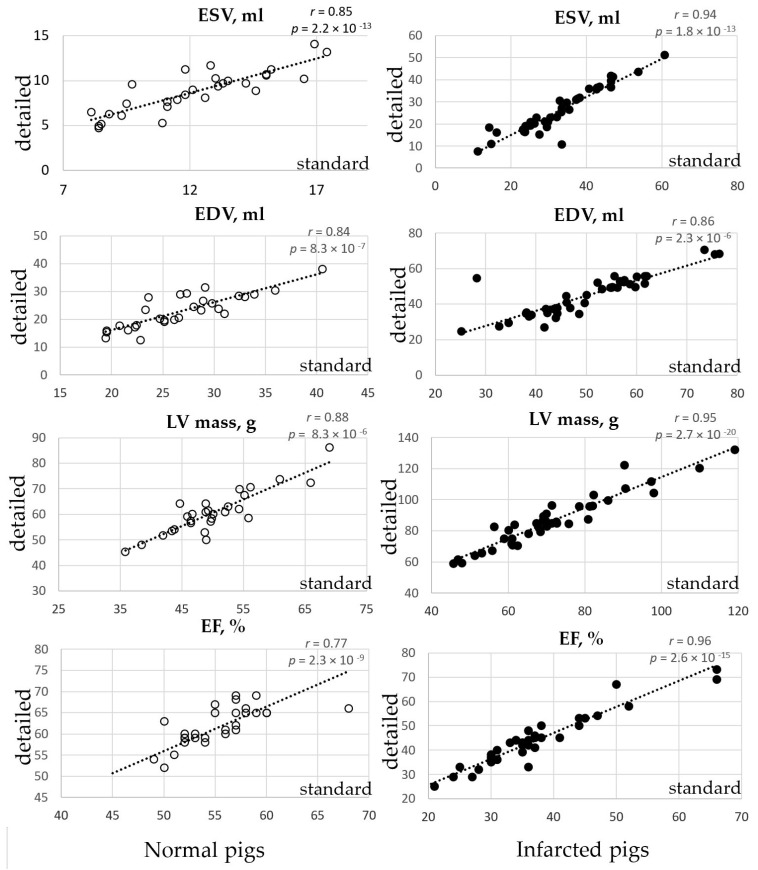
Correlations between the standard and detailed methods of the major volumetric measurements of the left ventricle in the normal (**left** column) and infarcted pigs (**right** column). Standard measurements are shown on the x-axis, and detailed measurements are shown on the y-axis of all scatter plots.

Twenty-nine healthy Yucatan mini pigs were subjected to CMRI study. The average weight of animals at the time of CMRI was 32 ± 2 kg. The average heart rate during imaging was 87 ± 23 beats per minute (bpm). In the normal pig hearts, there was a significant difference in LV volumetric assessments depending on the measurement method. Except for the stroke volume, a very high statistically significant difference was shown for all measured parameters, including LV mass, the LVV, the ejection fraction as well as all indices derived from LV mass (*p* < 0.001, Table 1). Specifically, estimation of the LV chamber volumes in systole and diastole was 28% and 15%, respectively, and smaller if the detailed tracing method was used for the ESV and EDV calculations (*p* = 2.18 × 10^−13^ and 8.34 × 10^−7^, respectively). Estimation of the LV mass was 21% higher in the detailed method in comparison with the clinically preferred standard tracing technique (*p* = 8.33 × 10^−6^). The ejection fraction measurement in the pig heart was 11% higher in the detailed method (*p* = 2.28 × 10^−9^). All indices derived from LV mass (LV mass/Body weight, LV mass/ESV, LV mass/EDV) were affected much stronger by the measurement method: 23% (*p* = 1.79 × 10^−8^), 77% (*p* = 5.39 × 10^−13^) and 50% (*p* = 6.52 × 10^−10^), respectively. There was a strong correlation between all volumetric measurements obtained with the standard and detailed methods (Table 1, Figure 3).

### 3.2. Volumetric LV Analysis in the Infarcted Pigs

A total of 40 CMRI exams were conducted at different time points after the modeling of myocardial infarction (MI). The average weight of animals at the time of CMRI was 36 ± 5 kg. The average heart rate during imaging was 83 ± 21 bpm. In the infarcted pig hearts, the differences in the LVV obtained by the two methods were also well pronounced for all volumetric measurements, with the exception of the SV, CO and infarct size (Table 2). LVV estimations were 21% (ESV) and 11% (EDV) smaller with the detailed method than with the standard (*p* = 1.80 × 10^−13^ and 2.32 × 10^−6^, respectively). If the detailed method was used, LV mass appeared to be 21% higher (*p* = 2.74 × 10^−2^) and the ejection fraction was 19% higher (*p* = 2.59 × 10^−15^). LV indices (LV mass/body weight, LV mass/ESV, and LV mass/EDV) were affected even more by the measurement method: the increase in the indices was 18% (*p* = 1.53 × 10^−9^), 57% (*p* = 6.11 × 10^−8^) with the standard method, and 42% (*p* = 3.52 × 10^−9^) with the detailed method, respectively. Infarct size measurements were not affected by the LV tracing method (*p* = 0.19). The correlation between the standard and detailed methods of LV tracing was very high for all studied parameters (Table 2, Figure 3). The correlations between the standard and detailed methods of measurements were found stronger for the infarcted pig cohort. The data spread around the means were 2–5-fold larger for all measured parameters in the infarcted pigs in comparison with the same parameters obtained in the healthy animals, independent of the measurement method.

The means and standard deviations for the main volumetric measurements of the pigs’ hearts at 2, 4, 8, 12, and 16 weeks after MI are shown in Table 3. There was a significant negative LV remodeling detected by both measurement methods (standard and detailed) as described by the increased LVV and LV mass from 2 until 16 weeks after MI. Specifically, the ESV increased from week 2 to week 16 from 26.9 ± 8.3 mL to 39.7 ± 7.9 mL (48% increase detected with the standard method, *p* = 0.02); the ESV increased from week 2 to week 16 from 21.8 ± 7.6 mL to 26.9 ± 13.6 mL (23% increase if the detailed method was used, *p* = 0.28). The EDV increased from week 2 to week 16 from 45.0 ± 10.3 mL to 55.5 ± 7.4 mL (23% increase detected with the standard method, *p* = 0.04); the EDV increased from week 2 to week 16 from 41.1 ± 10.4 mL to 45.5 ± 9.7 mL (11% increase if the detailed method was used, *p* = 0.27). LV mass also increased in all infarcted hearts. Specifically, if estimation was done with the standard tracing technique (Table 3), the increase in LV mass was 25%, from 66.9 ± 11.8 g to 83.6 ± 11.9 g (*p* = 0.02). If estimation was done with the detailed tracing technique, the increase in LV mass from 2 till 16 weeks after MI was 19%, from 81.6 ± 13.0 g to 97.2 ± 6.3 g, *p* = 0.002.

The heart contractile function decreased over time after the MI modeling in pigs; this is presented by a 30% decrease in the EF from 40.9 ± 8.5% to 28.8 ± 5.2% if measured with the standard method (*p* = 0.005), while the EF decreased from 47.5 ± 9.7% to 36.3 ± 6.4% if measured with the detailed method (24% change from week 2 to week 16, *p* = 0.02). A decrease in stroke volume over time was also statistically significant (*p* = 0.03 for both methods). Changes in cardiac output in the infarcted pigs’ hearts over time were not statistically significant (*p* > 0.05).

Papillary muscle mass was assessed in the same animals before and 2 weeks after myocardial infarction (*n* = 16). The papillary muscle mass in the normal pigs was 10.6 ± 5.4 mg; the papillary muscle mass increased after MI to 15.1 ± 5.7 mg, and the difference between the normal and infarcted papillary mass was statistically significant (*p* = 0.03, paired *t*-test, two-tailed). Despite the increase in the absolute value of the papillary muscle mass after infarction, the relative mass of the papillaries to the whole LV mass did not change after MI, because of the LV hypertrophy of the left ventricle after injury.

### 3.3. Comparison of LV Mass Assessment In Vivo and Ex Vivo

**Figure 4:**
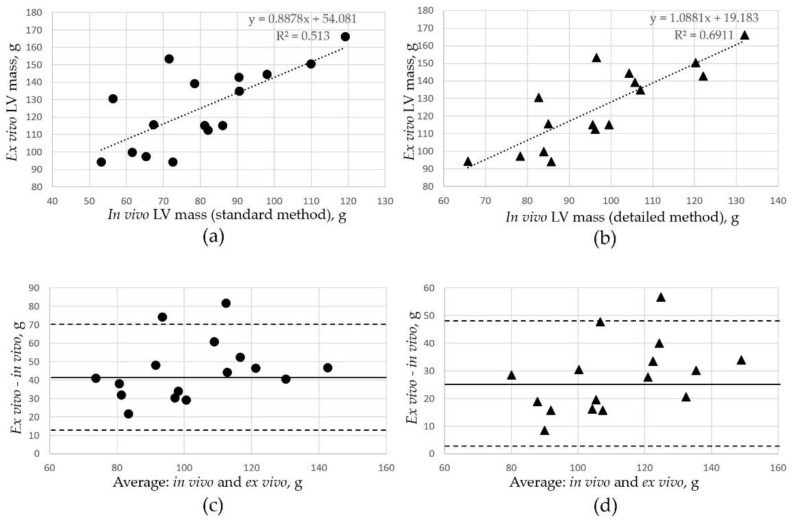
Comparison of LV mass estimation with *in vivo* MRI and *ex vivo* after heart extraction. (**a**) Relation between in vivo LV mass estimated with the standard method and post-mortal *ex vivo* LV mass. (**b**) Relation between *in vivo* LV mass estimated with the detailed method and post-mortal *ex vivo* LV mass. (**c**) Bland–Altman plots of *in vivo* LV mass estimated with the standard method versus LV mass obtained *ex vivo*. (**d**) Bland–Altman plots of *in vivo* LV mass estimated with the detailed method versus LV mass obtained *ex vivo*. Solid lines represent the mean difference of each parameter assessment by different reviewers; the broken lines mark the 1.96% standard deviation of the mean differences in measurements.

The mean ± SD value of *ex vivo* LV mass in the Yucatan minipigs was 125 ± 23 g, as measured in 16 hearts after extraction and right ventricle removal. The mean in vivo LV mass in the same animals was 80 ± 19 g if assessed using the standard endocardium tracing technique and 98 ± 18 g if the detailed method was used. The differences between *in vivo* and *ex vivo* LV mass measurements were statistically significant (*p* = 1.15 × 10^−6^ for the standard CMRI method and *p* = 0.0006 for the detailed technique). The *ex vivo* LV mass was strongly correlated with MRI-derived in vivo measurements of LV mass; the correlation coefficients were *r* = 0.72 in the standard/*ex vivo* pairs of measurements and *r* = 0.83 in the detailed/*ex vivo* measurements (Figure 4a,b).

Bland–Altman analysis (Figure 4c,d) showed a significant underestimation of the LV mass from the CMRI measurements with both the standard and the detailed tracing methods, with smaller differences when the detailed analysis was used. The mean difference between in vivo standard measurements and *ex vivo* assessments was 45 ± 23 g. The detailed LV tracing method showed closer results for *ex vivo* LV mass; the mean difference was 28 ± 13 g. 

### 3.4. Comparison of Volumetric Analysis between Reviewers

We compared the LVV measurements of the randomly selected CMRI cases conducted by two reviewers (AVN, experienced reviewer and KP, new reviewer) using the standard and detailed methods. The correspondence of the measurements between reviewers was high, as represented by the Pearson correlation and interclass correlation coefficients (Table 4). However, there was a statistically significant difference between measurement of the ESV, EDV and LV mass, while contractility results (specifically the EF) did not differ between the two reviewers. The difference was more apparent with the standard analysis. The interclass correlation coefficient (ICC) was used to assess the consistency of measurements made by the different CMRI reviewers (Table 4). The consistency was found in the assessment of LV mass between the two reviewers using the detailed method (ICC = 0.94), as well as in the EDV and EF (ICC = 0.90 and 0.92, respectively). The lowest consistency was found in tracing of the LVV in the systolic phase of the cardiac cycle using the standard method (ICC = 0.65).

**Figure 5:**
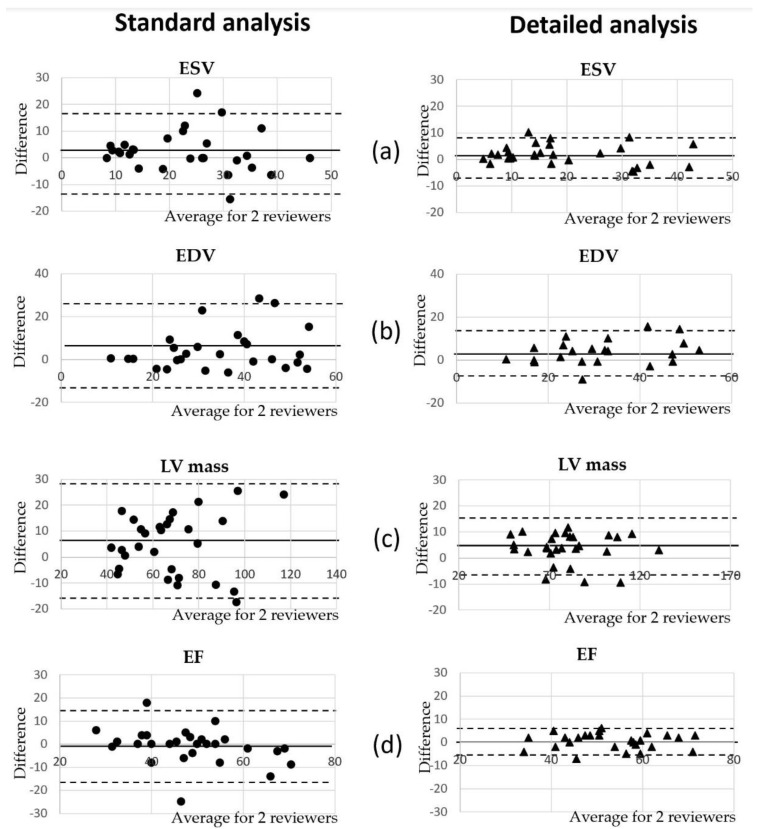
Bland–Altman analysis of the interobserver variability for two reviewers using the same Philips ISP software. The solid line represents the mean difference of each parameter assessment by the different reviewers; the broken lines mark the 1.96% standard deviation of the mean differences in measurements. The mean values obtained by the two reviewers are plotted in the x-axis; the differences between measurements of the two reviewers (reviewer 1–reviewer 2) are plotted on the y-axis. The panels (**a**–**d**) show interobserver variability in measurements of ESV, EDV, LV mass and EF, correspondingly.

Bland–Altman analysis was conducted to assess an agreement in measurements between the reviewers (Figure 5). The significant bias was found in the assessment of the LVV and LV mass by both methods, standard and detailed; however, the mean differences between the two reviewers were smaller when the detailed method of analysis was used. In the majority of CMRI cases, the LVV and LV mass were found to be smaller if assessed by a new reviewer. EF estimations were close between the two reviewers in both methods (Figure 5).

## 4. Discussion

The accurate assessment of the left ventricular mass and chamber volumes with CMRI is of diagnostic and prognostic importance for patients with LV hypertrophy [9,10,14,15], as well as for phenotyping animal models most often used in pre-clinical studies, such as the pig [4,16,21,22,23,24,25]. The present CMRI study conducted in healthy and infarcted minipig hearts showed significant differences in LVV and LV mass measurements obtained in vivo by two different methods of endocardium tracing. In the standard tracing method, the papillary muscles and the trabeculae were considered as a part of the LV chamber, while in the detailed analysis the papillary muscles and the trabeculae were counted toward the myocardial mass. Exclusion of the papillary muscles from the LV myocardium (as in the standard method) significantly reduced the calculated ejection fraction and increased LV chamber volumes. Conversely, inclusion of the papillary muscles and the trabeculae to the myocardial mass (as in the detailed method) increased the ejection fraction and decreased LV chamber volume values significantly.

The published literature data on the papillary mass in the pig heart vary. The study of Kirschbaum [11] showed that the papillary muscle mass in the hearts of Yorkshire-Landrace pigs was 4.7 ± 1.8 g and accounted for 4.5% of the total measured LV mass. The study of François et al. [4] counted papillary muscles mass as 7% of the LV in five pigs. In our study conducted on Yucatan minipigs, they had relatively larger papillary muscles in comparison with the Yorkshires; based on our data, papillary muscles added 21% to the LV mass in the healthy and infarcted pigs’ hearts (*p* < 0.001). Since the Yucatan minipigs are preferred for long-term studies, the significant papillary muscle mass should be taken into account. The detailed method of *in vivo* LV analysis showed closer results to the LV mass than *ex vivo*, which outlines the importance of the papillary muscles’ inclusion into the total LV mass. However, there was not an absolute correspondence between the LV mass measured by the detailed MRI method and the *ex vivo*. The main reason for larger LV mass measurements *ex vivo* was related to the large variability of the *ex vivo* data. In some cases, hearts were explanted not on the same day after the MRI exam, but at later time points. Those animals were part of the larger cell transplantation study, and the time of euthanasia was dependent on other considerations.

Pigs are the most often used large animal model for pre-clinical studies of cardiovascular devices and therapies. While publications on human patients have shown the importance of the detailed tracing method for the assessment of heart volumes in patients with hypertrophic cardiomyopathy [10,15], there is no uniform approach to the CMRI analysis of pig cardiac LVV and contractility. In publications of Gho et al. [21] and Natsumeda et al. [22], pigs’ papillary muscles and trabeculae were not excluded from the LV cavity volume. In Lopez et al. [23], the papillary muscles were excluded from the LV cavity volume based on the analysis of cine images and excluded from the myocardial mass based on the infarct size analysis. Many published CMRI studies involving pigs [16,24,25,26] do not even mention the endocardium tracing method, which brings confusion to the interpretation of results.

Since the papillary muscles accounted for almost a quarter of the total LV mass, this cannot be ignored. Inclusion or exclusion of these from the CMRI analysis might significantly affect the study results. The detailed method of endocardium tracing accounts for the large volume of the papillary muscles in the pig heart, and might be preferable in the analysis of LV mass. Our results are consistent with other publications that have shown that LV mass is determined most accurately when the papillary muscles and the trabeculae are included in the LV mass measurements in humans as well as in pigs [9,10,11,15]. Based on our data, the detailed LV tracing method showed a closer match of the LV mass to the *ex vivo* assessment; some mismatches between *in vivo* and *ex vivo* measurements might be explained by the large variability of the *ex vivo* data, with several outliers that had significantly larger LV mass assessed post-mortem much later after the CMRI exam.

The LV ejection fraction was significantly higher if assessed with the standard tracing method in normal as well as in infarcted hearts. The 11% absolute difference in the EF in normal subjects and the 19% difference in infarcted hearts is significantly larger than the effect size of most cardiac therapies [27]. Interestingly, that stroke volume did not differ between the measurement techniques. This suggests that, despite evident differences in LV volume assessed by the two different approaches, both could be used for the assessment of mitral regurgitation.

In our study, a strong correlation between all volumetric measurements in the pig heart was obtained with the standard and detailed methods of endocardium tracing. However, correlation coefficients in LVV measurements in the normal pigs (Pearson correlation ranged from 0.7 to 0.88) were lower than in the infarcted animals (Pearson correlation ranged from 0.75 to 0.98). This might be related to the difficulties in the endocardium tracing of the normal heart, because large papillary muscles almost completely obscure the LV chamber at the end-systole, and also because of the partial volume averaging of trabeculations. The diastolic contours, using the standard method, are easier to define in infarcted hearts because they are characterized by larger LV chamber volumes and thinner LV walls as the result of MI, which makes LV chamber tracing less challenging and correlations between the standard and detailed measurement methods higher. On the contrary, it is more difficult to contour the diastolic images in both the normal and infarcted hearts with the detailed method, due to the partial volume averaging of papillary muscles and trabeculations. The data spread around the means were 2–5-fold larger for all measured parameters in the infarcted pigs in comparison with the same parameters obtained in the healthy animals, independent of the measurement method. Larger data variability might be related to the individual responses to myocardial injury of the different subjects, as well as differences in the time points after MI.

The detailed method of endocardium tracing showed the highest interoperator consistency for the assessment of LV mass and the ejection fraction between two blinded reviewers using the same software in analysis. Our work is consistent with previously published studies on human patients and large animals comparing left ventricular volume calculations by the detailed and standard methods [11,14,15]. The detailed method of myocardial tracing requires about 30% longer time in comparison with the standard analysis (6 min 30 s and 9 min 30 s, respectively). Though the present study adds to the existing and partly controversial literature on LV assessment in animal models, the results cannot be immediately transferred to a human setting.

## 5. Conclusions

Significant differences in left ventricle volumetric and functional measurements result from the CMRI measurement method, whether papillary and trabecular muscles are included as part of LV mass or the LV cavity volume. The conclusions of this work are the following:

1. The standard clinical CMRI approach of endocardial tracing that excludes the papillary muscles and the trabeculae from LV mass underestimates ventricular mass and the LV volume in pigs due to the relatively large papillary muscles.
2. The detailed method of endocardium tracing accounts for the large volume of the papillary muscles in the pig heart, and despite the minor mismatch with the actual LV mass measured *ex vivo*, the detailed method might be preferable for the analysis of LV mass.
3. The papillary muscles add approximately 21% to LV mass in normal and infarcted hearts of Yucatan minipigs.
4. The detailed method of tracing all papillary muscles as part of LV mass requires ~30% more time for analysis in comparison with the standard method but provides better accuracy.
5. The detailed method of endocardium tracing showed better interobserver consistency in the assessment of LV mass and the ejection fraction.

## Figures and Tables

**Table 1:** Volumetric analysis comparison of the normal hearts of the Yucatan minipigs using the standard method and the detailed method (*n* = 29).

Measurement	Standard Method	Detailed Method	Mean Relative Difference	*p* Value	Pearson Correlation Coefficient
ESV, ml	12.2 ± 2.7	8.8 ± 2.4	−28%	2.18 × 10^−13^	0.85
EDV, ml	26.8 ± 5.3	22.9 ± 6.2	−15%	8.34 × 10^−7^	0.84
SV, ml	14.8 ± 3.1	14.2 ± 4.4	−4%	0.19	0.84
LV mass, g	50.1 ± 7.3	60.7 ± 8.7	+21%	8.33 × 10^−6^	0.88
CO, L/min	1.3 ± 0.3	1.2 ± 0.4	−8%	0.04	0.81
EF, %	55.0 ± 4.1	61.2 ± 6.0	+11%	2.28 × 10^−9^	0.77
LV mass/Body weight	0.0013 ± 0.0002	0.0016 ± 0.0002	+23%	1.79 × 10^−8^	0.7
LV mass/ESV	3.5 ± 1.0	6.2 ± 1.8	+77%	5.39 × 10^−13^	0.86
LV mass/EDV	1.6 ± 0.4	2.4 ± 0.7	+50%	6.52 × 10^−10^	0.79

Data are shown as mean ± standard deviation. *p* values are shown as the result of a paired *t*-test (two-tailed).

**Table 2:** Comparison of the volumetric measurements of the infarcted hearts of Yucatan minipigs using the standard method and the detailed method (*n* = 40).

Measurement	Standard Method	Detailed Method	Mean Relative Difference	*p* Value	Pearson Correlation Coefficient
ESV, ml	33.3 ± 11.1	26.2 ± 10.2	−21%	1.80 × 10^−13^	0.94
EDV, ml	50.2 ± 11.8	44.8 ± 11.5	−1%	2.32 × 10^−6^	0.86
SV, ml	17.2 ± 5.1	18.4 ± 5.8	+7%	0.05	0.75
LV mass, g	71.5 ± 16.4	86.5 ± 17.0	+21%	2.74 × 10^−2^	0.95
CO, L/min	1.4 ± 0.6	1.5 ± 0.5	+7%	0.08	0.86
EF, %	35.5 ± 10.7	42.1 ± 11.9	+19%	2.59 × 10^−15^	0.96
LV mass/Body weight	0.0017 ± 0.0005	0.002 ± 0.0005	+18%	1.53 × 10^−9^	0.94
LV mass/ESV	2.1 ± 0.9	3.3 ± 2.0	+57%	6.11 × 10^−8^	0.9
LV mass/EDV	1.2 ± 0.3	1.7 ± 0.6	+42%	3.52 × 10^−9^	0.9
Infarct size, % to LV	9 ± 4	10 ± 4	+11%	0.19	0.98

Data are shown as mean ± standard deviation. *p* values are shown as the result of a paired *t*-test (two-tailed).

**Table 3:** Volumetric measurements of the infarcted hearts of Yucatan minipigs at the different time points after MI using the standard and detailed methods.

Measurement	2 Weeks (*n* = 16)	4 Weeks (*n* = 7)	8 Weeks (*n* = 7)	12 Weeks (*n* = 6)	16 Weeks (*n* = 4)
Standard Method					
ESV, ml	26.9 ± 8.3	31.8 ± 11.9	31.6 ± 9.8	45.0 ± 11.4	39.7 ± 7.9
EDV, ml	45.0 ± 10.3	47.1 ± 9.6	48.3 ± 10.4	63.9 ± 12.8	55.5 ± 7.4
SV, ml	18.1 ± 3.9	15.3 ± 6.8	16.7 ± 6.6	18.8 ± 5.7	15.7 ± 1.2
LV mass, g	66.9 ± 11.8	86.2 ± 17.1	81.4 ± 26.3	71.1 ± 6.0	83.6 ± 11.9
CO, L/min	1.5 ± 0.5	1.3 ± 0.7	1.5 ± 0.6	1.5 ± 0.6	1.3 ± 0.5
EF, %	40.9 ± 8.5	33.6 ± 15.4	35.6 ± 11.7	30.0 ± 8.2	28.8 ± 5.2
Detailed Method					
ESV, ml	21.8 ± 7.6	25.3 ± 12.1	25.3 ± 7.8	36.8 ± 11.3	26.9 ± 13.6
EDV, ml	41.1 ± 10.4	39.8 ± 11.0	45.7 ± 8.3	57.2 ± 13.6	45.5 ± 9.7
SV, ml	19.2 ± 4.5	14.5 ± 5.1	20.4 ± 8.8	20.3 ± 6.1	16.1 ± 2.1
LV mass, g	81.6 ± 13.0	83.2 ± 18.6	96.5 ± 27.8	86.2 ± 10.0	97.2 ± 6.3
CO, L/min	1.5 ± 0.5	1.2 ± 0.6	1.8 ± 0.5	1.6 ± 0.7	1.3 ± 0.5
EF, %	47.5 ± 9.7	38.6 ± 16.4	44.4 ± 14.1	36.0 ± 9.5	36.3 ± 6.4

Data are shown as mean ± standard deviation.

**Table 4:** Correlations in assessment of the pig LVV and contractility between two reviewers using the standard and detailed methods (*n* = 28).

Measured Parameters	Main Relative Difference between Reviewers Using the Standard Method	Main Relative Difference between Reviewers Using the Detailed Method	Standard Method Pearson Interclass Correlation Correlation		Detailed Method Pearson Interclass Correlation Correlation	
ESV	9% *	8%	0.76	0.65	0.95	0.85
EDV	11% **	9% *	0.83	0.77	0.93	0.90
LV mass	7% **	5% *	0.83	0.83	0.96	0.94
EF	2%	1%	0.81	0.81	0.94	0.92

Notes: * marks the statistically significant difference between measurements of the same CMRI cases conducted in the Philips ISP software with *p* < 0.05 (paired *t*-test, two-tail); ** for *p* < 0.01.

## Data Availability

The datasets used and/or analyzed during the current study are available from the corresponding author on reasonable request.
